# Clinical Holistic Medicine: Holistic Sexology and Acupressure Through the Vagina (Hippocratic Pelvic Massage)

**DOI:** 10.1100/tsw.2006.337

**Published:** 2006-03-07

**Authors:** Søren Ventegodt, Birgitte Clausen, Hatim A. Omar, Joav Merrick

**Affiliations:** ^1^ The Quality of Life Research Center, Teglgårdstræde 4-8, DK-1452 Copenhagen K, Denmark; ^2^ Research Clinic for Holistic Medicine, Copenhagen, Denmark; ^3^ Nordic School of Holistic Medicine, Copenhagen, Denmark; ^4^ The Scandinavian Foundation for Holistic Medicine, Sandvika, Norway; ^5^ Vejlby Lokalcenter, Vejlby, Denmark; ^6^ Section of Adolescent Medicine, University of Kentucky, Lexington, Israel; ^7^ National Institute of Child Health and Human Development, Faculty of Health Sciences, Ben Gurion University, Beer-Sheva, Israel; ^8^ Center for Multidisciplinary Research in Aging, Faculty of Health Sciences, Ben Gurion University, Beer-Sheva, Israel; ^9^ Office of the Medical Director, Division for Mental Retardation, Ministry of Social Affairs, Jerusalem, Israel

**Keywords:** quality of life, QOL, philosophy, human development, holistic medicine, holistic health, urine incontinence, vulvodynia, chronic pain, pain during intercourse, orgasmic potency, sexuality, ethics, vaginal acupressure, Denmark

## Abstract

Many gynecological and sexological problems (like urine incontinence, chronic pelvic pains, vulvodynia, and lack of lust, excitement, and orgasm) are resistant to standard medical treatment. In our work at the Research Clinic for Holistic Medicine in Copenhagen, we have found that vaginal acupressure, or Hippocratic pelvic massage, can help some of these problems. Technically, it is a very simple procedure as it corresponds to the explorative phase of the standard pelvic examination, supplemented with the patient's report on the feelings it provokes and the processing and integration of these feelings. Sometimes it can be very difficult to control the emotions released by the technique, i.e., regression to earlier traumas from childhood sexual abuse. This review discusses the theory behind vaginal acupressure, ethical aspects, and presentation of a case story. This procedure helped the patient to become present in her pelvis and to integrate old traumas with painful emotions. Holistic gynecology and sexology can help the patient to identify and let go of negative feelings, beliefs, and attitudes related to sex, gender, sexual organs, body, and soul at large. Shame, guilt, helplessness, fear, disgust, anxiety, anger, hatred, and other strong feelings are almost always an important part of a sexual or functional problem as these feelings are “held” by the tissue of the pelvis and sexual organs. Acupressure through the vagina/pelvic massage must be done with great care by an experienced physician, with a third person present, after obtaining consent and the necessary trust of the patient. It must be followed by conversational therapy and further holistic existential processing.

## INTRODUCTION

Sexology is the medical specialty concerned with sexual dysfunctions. The major breakthrough in this field was made by Masters and Johnson in the middle of the last century, mapping the human sexual functions and dysfunctions[1,2]. William Howell Masters (1915–2001) was a gynecologist and Virginia Eshelman Johnson (1925–) a psychology researcher. They teamed up in 1957 to study human sexuality. Before them, in the late 1940s and early 1950s, Alfred C. Kinsey (1894–1956) had published two surveys of modern sexual behavior, “Sexual Behavior in the Human Male” and “Sexual Behavior in the Human Female”, which founded the groundwork for Masters and Johnson's work. Instead of asking people about their sexual activities, as Kinsey had done, Masters and Johnson observed sexual activity in the laboratory. They developed tools and techniques for accurately measuring the physical responses of 700 men and women during masturbation and intercourse. They published their findings in the book *Human Sexual Response* in 1966[1]. This book was well received by the general public, even though it was intended for the medical community, since the mechanics of sex had so far been a mystery. Masters and Johnson were the first to identify and describe the human sexual response cycle, which opened up the more effective treatment of sexual dysfunction and dissatisfaction with sexual activity presented as a natural and healthy human trait.

Masters and Johnson afterwards published *Human Sexual Inadequacy* in 1970[2]. This book discussed common problems such as impotency and premature ejaculation, and how to treat them. This work was the key in the development of sex therapy and together they opened a clinic in St. Louis for the treatment of sexual problems.

## HOLISTIC SEXOLOGY

The most profound theory for sexuality seems to be the theory of the anima and animus (the inner man or woman) of Carl Gustav Jung (1875–1961)[3,4]. Holistic sexology aims to take the established knowledge on sexology into an existential perspective, including the sphere of existential dimensions and problems[5,6] in the treatment of sexual and gynecological problems[7]. Existential dimensions are needed in this work because the sexual and gynecological problems are symptoms of unsolved existential problems, where the patient’s inner potentials for healing his/her own life, body, and existence are not mobilized. The reason that standard treatments do not work on some patients is not obvious. Often, there are hidden and severe traumas from violence or sexual abuse in the past, and these negative emotions are held by the pelvic tissues and organs. Studies from different western countries indicate an incidence of about 15% of girls being assaulted sexually in childhood[8,9,10] and many of these girls are likely to demonstrate severe pelvic problems in their youth. Sexual and gynecological problems resistant to standard therapy are typically problems with acceptance of own sex and sexuality, which do not have to originate from abuse. As originally suggested by Masters and Johnson, they can be a result of not having received the loving acceptance and touch needed in childhood[2,11]. It is obviously important that borders are not violated, but it is just as important that the father give the contact and acceptance the child needs as part of her infantile and undeveloped sexuality[7].

The Hippocratic (Hippocrates, 460–377 BCE) physician was aware of these diseases and his treatment included different physical procedures focused on the female pelvis, like smoking the vagina and massaging the pelvis[12]. The reason why these treatments were later condemned are debated; some authors find it a form of sexual abuse of the woman by the medical profession with an insufficient ethic[13]. Maybe there has been a regrettable crisis in the ethical standard of the average physician on entering modern-day commercial medicine, where power and money often seem more important for the physician than care for the patient. In holistic medicine, the physician and his/her patient are almost always very close and ethics are a subject of utmost importance (see the discussion). When it comes to the practice of pelvic massage, we might be at the essence of medical ethics and the ability to perform this procedure might have been the very reason why Hippocrates invented his strict medical ethics in the first place.

The traditional techniques of acupressure though the vagina has been tested and developed at the Research Clinic for Holistic Medicine in Copenhagen, and has been discussed with members of the International Society of Holistic Health and presented at the Second International Conference on Holistic Health, April 22–24, 2005 in Oslo (Sundvollen), Norway. The comments and critique have been integrated in the present paper.

Many chronic patients need holistic existential healing or healing of the wholeness of the person on the deepest level of their existence in order to become better. Before we continue, therefore, let us take a look at holistic medicine and the concepts of existential healing.

## THE BASIS FOR CLINICAL HOLISTIC MEDICINE

The life mission theory[5,11,14,15,16] is based on the philosophy that everyone has a purpose in life or talents. Happiness comes from living this purpose and succeeding in expressing the core talent in life. To do this, it is important to develop as a person into what is known as the natural condition or a condition where the person knows himself and uses all his efforts to achieve what is most important for him. The theory of talent[5] states that we have three major talents in life called purpose, consciousness, and gender. In relation to this paper, these dimensions may simply be love, power, and sex. Gender and sexuality are fundamental dimensions of human existence, which must be in a sound, natural, and undenied state for the person to live and function naturally and in full power.

The holistic process theory of healing[18,19] and the related theories for salutogenesis[20,21], meaning of life[22], and quality of life[23,24,25] found that the return to the natural state of being is possible whenever the person gets the resources needed for existential healing. The resources needed are holding in the dimensions of awareness, respect, care, acknowledgment, and acceptance with support and processing in the dimensions of feeling, understanding, and letting go of negative attitudes and beliefs. The preconditions for holistic healing to take place are trust and the intention that the healing *will* take place. Existential healing is not a local healing of any tissue, but a healing of the wholeness of the person, making him much more resourceful, loving, and aware of himself, his own needs, and wishes. In letting go of negative attitudes and beliefs, the person returns to a more responsible existential position with an improved quality of life. The philosophical change taking place when the person is healing is often a change towards preferring difficult problems and challenges, instead of avoiding difficulties in life[26,27,28,29,30,31,32,33]. The person who becomes happier and more resourceful often also becomes more healthy, more talented, and more able to function[34,35,36].

Sexual problems are found in four major forms: lack of libido, lack of arousal and potency, pain and discomfort during intercourse, and lack of orgasm[2]. It is possible to work with a holistic approach to sexology in the clinic in order to find and repair the negative beliefs, repressions of love, and lack of purpose of life, which seemingly are the core to problems like arousal, potency, and pain with repression of gender and sexuality[2,6,7,37,38]. The theory of talent[5,6] seems to be relevant for understanding human sexuality. It is highly important not to focus on the gender and genitals in order to understand the patient's sexual problems, because many problems related to sex can be solved on the level of the whole person[2,6,7,37,38]. But as important as it is not to focus there, it is also essential not to neglect the body and the feelings connected to it. Shame, guilt, helplessness, fear, and other strong feelings are almost always an important part of a sexual problem[2,7].

## ACUPRESSURE THOUGH THE VAGINA

Thousands of women have problems related to their pelvis and its organs, dominated by sufferings of the sexual organs, problems of the urinary tract, the locomotor system, and the intestines[39]. Another large group of patients have "nonanatomic" pelvic pains and discomforts of presumably psychosomatic nature, which often are very difficult to treat with biomedicine, but which seem to react better to psychosomatic treatments[40,41]. We therefore urgently need new treatment tools for this broad range of problems, from urine incontinence, bleeding, and hormonal disturbances; unwanted childlessness; sexual problems like pain during intercourse; primary vulvodynia; or low ability to feel lust, pleasure, sexual excitement, and/or to reach sexual climax; to non-inflammatory perineal and anal pains and discomforts like idiopathic aches (primary pruritus). From a holistic medical perspective, the problems are often caused by unsolved emotional problems that have been repressed into the pelvis and its organs. The emotional problems are related to negative beliefs about self, gender, body, organs, and sexuality.

Judging from clinical experience from the Research Clinic for Holistic Medicine in Copenhagen treating 20 patients with the 10 different problems mentioned above with holistic sexology (acceptance through touch and, when necessary, also vaginal acupressure), such problems can often be solved through healing the old wounds of the body and soul in holistic existential, gynecological, and sexological therapy. The healing process has, as in all other holistic therapy, three obligatory steps that we summarize with the words: feel, understand, and let go[18,19,42]. First the emotions have to be felt again; we call this phase "putting feelings onto the body". Then the patient has to find words, i.e., verbalize the emotions and understand where the problems are coming from; we call this "putting words on the feelings". Last but not the least, the person healing has to let go of the negative attitudes and decisions that were made when the trauma happened; we call this "putting consciousness in the words". In the clinical work, we use the therapeutic staircase that gives us the best assurance that we do not use a more invasive and potentially dangerous technique than necessary[43]. Acupressure through the vagina always builds on earlier sessions of acceptance through touch, which again come after sessions of emotional healing, trust, and holding, and always begin with "love and care" for the patient. This staircase is also used at the Nordic School of Holistic Medicine for training the holistic physician to use the techniques from the holistic medical toolbox (see Fig. 1).

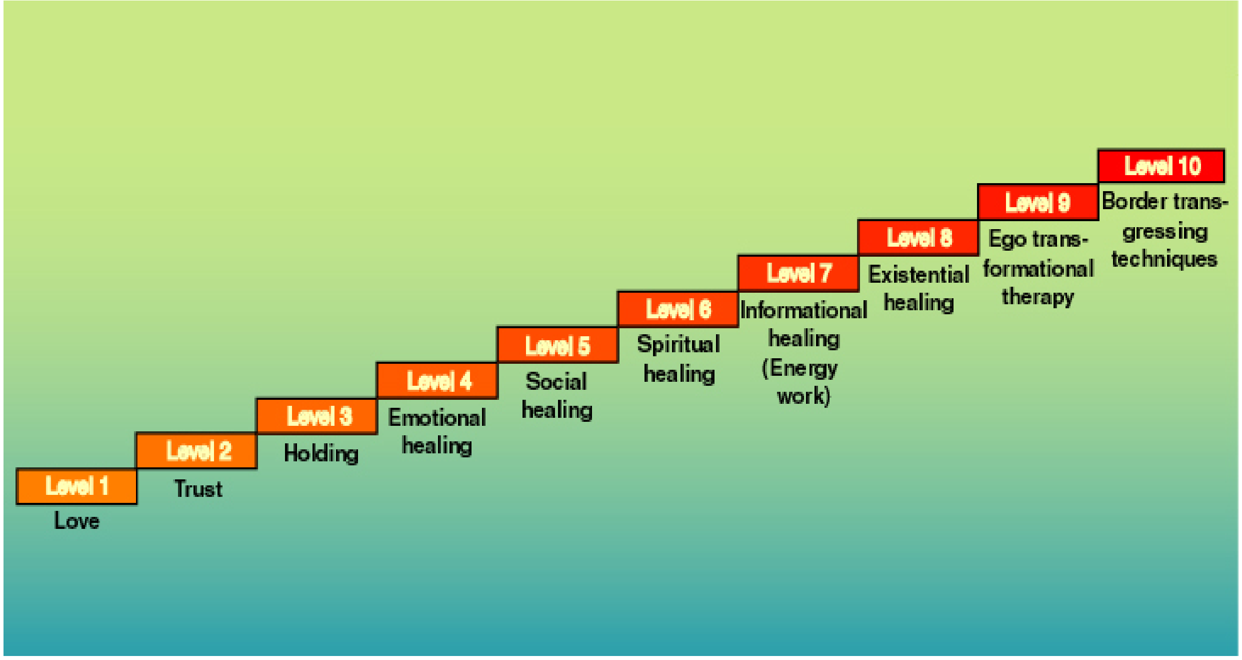

**FIGURE 1.** The therapeutic staircase of holistic medicine[43]. In therapy, as well as in the training of the therapist, one step must be accomplished before the next step is used. Please take note that vaginal acupressure is a level-8 technique in this system. Level 8 is the most difficult level in the advanced holistic medical practice. Most holistic therapists work at level 3 and 4 and most problems related to health, quality of life, and ability can be solved at this level.

This knowledge of healing life (improving health, quality of life, and ability in one integrated movement) is well known and described in a number of books from the cradle of medical sciences on the island of Cos around 300 BCE, known as Corpus Hippocraticum. Hippocrates (460–377 BCE) was held to be the best physician of his time and father of the first scientific system of holistic healing described in numerous books. It is interesting that massaging the pelvis through its openings was an acknowledged method in ancient Greece[12] and in use throughout Europe for centuries[13]. This necessitated the very stringent medical ethics practice that was founded by Hippocrates probably, as mentioned above, with the purpose so that he himself and his many pupils could give this kind of treatment. Massage of the pelvic structures of a woman through the vagina and anus could, among other things, heal disturbances in the woman's energy system known as a disease called "hysteria" from the Greek word for uterus, *hystera*. The treatment was in use in most of the western world until the industrial revolution, where it was condemned as pornographic and hence no longer an acceptable medical treatment.

After the sexual revolution in the 1960s and 1970s, we now have a more relaxed attitude towards the body and sexuality, and some therapists work again with this kind of therapy through the vagina and anus either by using their hand to cure sexual and other problems[44], or by using a vibrant penis substitute (a "dildo") to cure incontinence[45] or orgasmic problems[46]. The Danish physiotherapist Birgitte Bonde reports that one to six sessions with the vibrator can help many incontinent women who are not sufficiently helped by the standard program of training the pelvic floor[45]. The rationale for the use of the vibrator is that the woman cannot get in contact with their own pelvis, as they "cannot find their pelvic floor", presumably because they have completely eradicated some of the pelvic structures from their inner description of their own body.

There are several different forms of pelvic massage/vaginal acupressure (see Table 1) used for different purposes with as many philosophies about its mechanisms. Most therapists intend to raise the energies in the meridians found in the Chinese system[44], hence the name "vaginal acupressure" for the technique, often used for healing chronic pains in the pelvis or genitals, and treating the highly inconvenient pattern of frequent reinfection of the urinary system. Other therapists intentionally liberate the sexual energies with sexual stimulation, according to the old Indian Tantric tradition, in order to teach the woman to contain and handle her sexual energies[47]. We find it important to note here that the physician, under no circumstances, should attempt to stimulate the woman to an orgasm in order to avoid a sexual situation. Others work with confrontational therapy to heal traumas of incest and rape by integrating the bioenergetic system of Lowen[48], Reich's sexual therapy[49], and the gestalt therapeutic tradition[50] to be able to release all negative emotions and other problems caused by the prior sexual violation or neglect. In our clinic in Copenhagen, we have also found it useful to help women heal what we call the "sex – love split", making them having two partners, one for sex and one for love, and being unhappy with not being able to have love and sex with the same person.

All the above-mentioned practices have in common that they seek to help the patient notice the tensions and blockages in the pelvic region, and the parallel attitudes fragmentizing the patient's life. When the patients confront and integrate the repressed painful feelings that created them, they develop a new and more positive understanding of life, love, feelings, and sexuality.

It is clear that elements of acupressure through the vagina must be adjusted to the needs of the patient. A patient with chronic bladder infection and a patient with chronic pain in the pelvis or the sexual organs (primary vulvodynia) should be treated differently. It is important to always go for the lesser level of treatment that can solve the problem and the least provocative or painful of methods must be tried before more "embarrassing" methods are taken into use. We recommend that simple antibiotics are used to alleviate some of the problem and only problems that cannot be efficiently treated with such drugs should be handled with the emotionally challenging procedure of acupressure through the vagina — except in the cases where the patient does not want to take the drugs for personal, political, religious, or other reasons. If the physician believes several methods to be equally efficient, he should always tell the patient about the alternative treatments and respect the patient's choice. Holistic existential therapy will be more work for the physician and, in the end, less money paid by the patient as health problems are often solved permanently with holistic existential therapy.

**TABLE 1**
**Different Forms of Traditional Pelvic Massage/Acupressure Through the Vagina Organized According to the Emotional Core Problems***

| Dominant Emotional Problem | Style of Work | Corresponding Chakra | Primary Inspiration |
|---|---|---|---|
| Anxiety, insecurity, | Acupressure | Root | Chinese medicine |
| Physical pain related | through the | | |
| to kidney and urinary | vagina, pelvic massage | | Hippocratic med. |
| tract and intestines | Meridian work | Root, All | |
| Incontinence, shame | Use of vibrator | Root, Hara | Modern sexology |
| | | | |
| Sexual and hormonal. Problems | Pelvic and anal | Hara, Root | Hippocratic medicine, Indian tantric tradition, Chinese medicine |
| Pains during intercourse | massage/ | | |
| Problems with lust, | acupressure | | |
| joy, excitement, and | through the vagina | | |
| orgasm, shame | and anus | | |
| Low self esteem, | Use of vibrator | Hara, Root | Modern sexology |
| Polarity problems | Raising energy | Hara, All | Jung's theory of anima and animus |
| Sexual energy work | circles | | |
| | | | |
| Relational problems | Pelvic and anal | Solar plexus | Hippocratic med. |
| Problems with men after incest, rape and other violations, hate, anger, shame, guilt | massage, acupressure through the vagina and anus | Solar plexus, All | Chinese medicine |
| | Controlled sexual abuse[43] | Solar plexus | Gestalt therapy |
| | | | |
| Problems with integrating | Pelvic and anal | Heart, Root, Hara | Hippocratic medicine Chinese medicine |
| Love and sexuality | massage, acupressure | | |
| "Sex love split", Adultery | through the vagina | | |
| Prostitution, Sexual | Direct sexual stimulation[43] | Hara, Heart, 3 eye | Indian tantric tradition |
| domination/submission | Use of role-plays | Heart, All | Gestalt therapy |

*The therapy must always be followed by thorough conversational therapy for full integration and performance must be according to ethical standard (see text). Interestingly classical western medicine, Chinese medicine, and Indian medicine seem to have used related techniques.

If a sexual problem can be solved with just giving acceptance to the body, there is no reason to approach the sexual organs. If just giving acceptance to the outside of the vulva is enough to solve the problem, there is no rationale for penetrating the vagina[7]. Often the feelings of guilt and shame that are the cause of the problem can be solved by the smaller process we call "acceptance through touch" and, in this case, it would be unethical to start with acupressure though the vagina. If conversational therapy can do the job, touching the vulva will be unethical. In every case, the physician must treat according to his or her best judgment. Medicine will always be an art and only the trained physician knows which tool to use with a patient as both intuition and experience is necessary for the decision.

## THE PROCEDURE OF VAGINAL ACUPRESSURE

Vaginal acupressure is technically the most simple procedure as it corresponds to the explorative phase of the classic pelvic examination, except that the purpose of the digital penetration is treatment and not examination. Vaginal acupressure is performed by placing the woman on the examining table in a relaxed position with free passage to the vagina (see Fig. 2). The physician penetrates the vagina with one or two fingers and presses systematically on the sore and tense areas in the pelvis. Most organs are accessible to the trained therapist. The position of the physician's hand must be so that only the structures that need to be touched are contacted (it is important that the clitoris is not touched unintentionally). The applied pressure is adjusted to the situation to optimize the therapeutic effect, as described by Marion Rosen[51]. The indication for using this procedure in the holistic medical clinic must always be the physician's understanding of the need of the patient for contact with the structures inside the pelvis. An attending nurse or another person must be present and give "holding" and support to the patient.

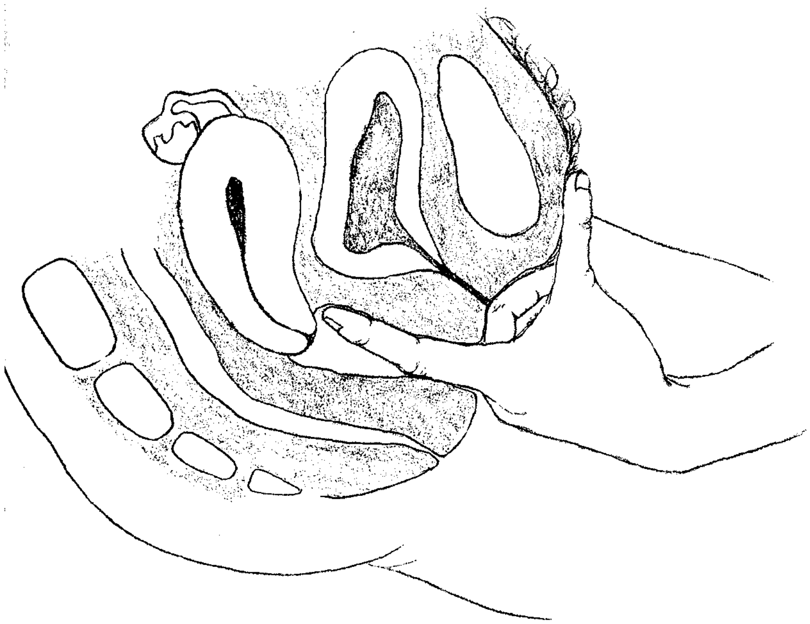

**FIGURE 2.** Sexual healing though the vagina by pressing on the tissues and helping the patient to identify and process the repressed feelings and old traumas held by the pelvic organs[47]. The vagina is penetrated with one or two fingers, and all the structures of the pelvis are systematically worked through. The patient is invited to open up to the feelings hidden in the tissues and these feelings are then processed in holistic existential therapy.

It is important to understand that the procedure of acupressure through the vagina is the same explorative part of the standard pelvic examination by a gynecologist, but in this case done so slowly that the woman can feel the emotions held by the different tissues contacted by the finger of the physician[38]. It can be used in combination with the pelvic examination and as the woman always will contact some feelings while examined in her vagina, the situation is really that every pelvic examination contains an element of acupressure through the vagina. Often the awakening of unpleasant feelings is very emotionally painful for the woman and if not taken care of by the physician/gynecologist, it will make the standard pelvic examination difficult for the woman, as many woman actually experience. Just ignoring the fact that the woman is a living human being reacting emotionally to the pelvic examination is not going to help the woman not to feel.

## ETHICAL ASPECTS

The procedure of acupressure through the vagina must be performed according to ethical standards. The holistic sexological procedures are derived from holistic existential therapy (which involves reparenting, massage and bodywork), conversational therapy, philosophical training, healing of existence during spontaneous regression to painful life events (gestalts), and close intimacy without any sexual involvement.

In psychology, psychiatry, and existential psychotherapy[52,53], touch is often allowed, but a sufficient distance between therapist and client must always be kept, all clothes kept on, and it is even recommended that the first name is not taken into use to keep the relationship as formal and correct as possible[54]. The reason for this distance is to create a safety zone that removes the danger of psychotherapy leading to sexual involvement. In the original Hippocratic medicine[12], as well as in modern holistic existential therapy, such a safety zone is not possible because of the simultaneous work with all dimensions of existence, from therapeutic touch[55] of the physical body, feelings, and mind to sexuality and spirituality. Since Hippocrates, the fundamental rule has been that the physician must control his behavior, not to abuse his patient. The patients in holistic existential therapy and holistic sexology are often chronically ill and their situation often pretty hopeless, as many of them have been dysfunctional and incurable for many years or they are suffering from conditions for which there are no efficient biomedical cures.

The primary purpose of holistic existential therapy is to improve quality of life; secondary, to improve health and ability. The severe conditions of the patients and the chronicity is what ethically justify the much more direct, intimate, and intense method of holistic existential therapy, which integrates many different therapeutic elements and works on many levels of the patient’s existence and personality at the same time. Holistic sexology is holistic existential therapy taken into the domain of sexology. The general ethical rule is that everything that does not harm and will help the patient in the end is allowed (“first do no harm”). An important aspect of the therapy is that the physician must be creative and, in practice, invent a new treatment for every patient as Yalom has suggested[52,53]. To perform the sexological technique of acupressure through the vagina, the holistic sexologist must be able to control not only his/her behavior, but also his sexual excitement to avoid any danger of the therapeutic session turning into sexual activity. Most physicians can do the classic pelvic examination after their standard university training, but the vaginal acupressure we are discussing here in this paper can only be obtained through long training and supervision in order to reach a level where such a procedure can be performed.

Side effects of the treatment can be soreness of the genitals and periods of bad mood as old painful repressed material are slowly integrated. We have seen what we call an acute psychosis as a sexually abused woman confronted her most painful experiences, but she recovered in a few days without the use of drugs and this episode was an integral part of her healing. In fact, it was her therapeutic breakthrough. As it is possible that the patient can feel abused from transferences, it is extremely important to address this openly to prevent this situation. We recommend that the patient is contacted or followed up for 1–5 years to prevent and handle any potential long-term negative effects of the treatment. In spite of these problems, we have found the treatment with holistic existential therapy combined with the tool of vaginal acupressure to be very valuable for the patients.

## CASE STORY

The following case story from the Research Clinic for Holistic Medicine in Copenhagen and also the case of Anna[56] made us reinvent the method of vaginal acupressure. This index case was a cancer patient who did not heal in the therapy, although level 1–7 of the therapeutic staircase (see Fig. 1)[43] had been taken into use for several months, so instead of giving up on her, we (SV and BC) reinvented the level-8 technique of acupressure through the vagina. The patient was part of our cancer project where we try to induce spontaneous remissions in metastatic cancer[57]. She had opted not to receive surgery, chemotherapy, or radiation therapy, as she was dissatisfied with the less than 30% chance of surviving her cancer with biomedicine (this was the option given to her at the Department of Oncology at the University Medical Center, where she was diagnosed and offered treatment).

> **Female, 39 years, multiple sexual traumas in childhood and now metastatic breast cancer (excerpt from our chart)**
>
> 20th session at our clinic: The cancer is not healing judged from the size of the tumours. Her tumours in the right breast and in the armpit are still growing. The patient is remarkably difficult to get into the emotional process of healing, presumably because the repressed emotional pains from the childhood sexual traumas are too strong. We agree to try to send her back into the gang rape traumas from her youth by using tools of the next treatment level. After written consent we decided to use acupressure through the vagina. We combined the level 5 and level 8 of the therapeutic staircase by having several nurses present to optimise holding. Immediately after the penetration of the vagina, she regressed into being in one of the rape situation and she suffered unbearable emotional pain, which she this time succeed in confronting. For the first time she was able to confront what happened on an emotional level. Conversational therapy.

After the above session, there were several sessions of integrative conversational therapy. For the first time, she was able to enter the holistic process of healing in the sessions. The uncontrolled growth of her tumor stopped after the session above. It seemed that there was a connection between the emotional pain from the rape trauma with the growth of her cancer (which is in accordance with the holistic theory for cancer[57,58]). In this case, acupressure through the vagina did what less-intense holistic medical tools could not do for her. The acupressure sent her into the old emotional pain, helped her to integrate it, and thus heal her existence and maybe also her cancer. If she will survive the cancer, it looks like this session was the turning point. We followed up with her for 3 months and she felt good, but then she decided to move to South America to start a new life and we have not had contact with her since.

## DISCUSSION WITH ETHICAL CONSIDERATIONS

In all work with clinical holistic medicine, ethics is of utmost importance and when the physician penetrates (with his finger) the vagina of a patient with the intention of healing, we are at the most critical of situations. Hippocrates said: “First do no harm”, and acupressure through the vagina is potentially extremely harmful and we judge it to be one of the most difficult of the holistic medical tools to master. Three aspects of the physician’s behavior must be taken under careful consideration before the therapy begins:

- What is the intention of this treatment? Can the result be achieved in other ways? Does the physician have the required skills to perform the therapy? Is there a (written) consent from the patient? Have the all steps of the procedures been discussed thoroughly so that the patient knows exactly what to expect? Have preventative measures been taken to avoid later interpretation of the treatment as a violation of the patient? Does the physician have the proper insurance for this kind of work?
- During the therapy, it is of utmost importance that the physician and the patient remain in contact at all times, the physician must look the patient in the eyes in a relaxed way to ensure the patient that everything is going as it should. The nurse must give holding to the patient. Balance and contact are the keywords for smooth and trouble-free therapy. If the patient gets into emotional pains, this must be taken care of right away; if the patient unintentionally gets sexually exited, the physician must be trained to contain that without getting into sexual excitement him or herself. The physician must be trained to be able to control his own sexuality to such a degree that the healing of the patient is the sole focus of the physician's intention, and acupressure through the vagina must always be done under proper supervision.

- The effect of the therapy must always be measured. A small quality of life and health questionnaire like QOL1 and QOL5[59,60,61] administrated to the patient before, after, and years after the therapy is a must in this kind of therapy, so that the physician can be sure that he actually helps the patient, now and in the long term. It is easy to believe that the patient has been helped immediately after the completion of the therapy, but what is important is that the patient also finds that the therapy has been helpful years after it has ended. After each session, what has happened must be thoroughly discussed with the patient, and the patient-physician relationship must be cleared whenever there is a retraction or an emotional issue in the relationship.

It should always be remembered that holistic existential therapy and healing is not really a technique, but rather a gift of care or, in essence, love in an unselfish support of the patients. Touching the genitals of a patient with the intention of (sexual) healing cannot be successfully accomplished without the combination of love or intense care, and a high ethical standard. To say this very clearly, only the physician who has a heart and care can touch the patient for the sake of healing the patient. Without love, confidence, and skillful holding[5,38,39], the procedure will not work.

In holistic sexology work with patients, the physician must always be present as a human being. Often the physician doing this kind of work will have qualms and concerns, and must be extremely cautious and conscientious when breaking one of the toughest taboos in the medical world, namely sex. It is severely frowned on to touch the female private parts if it is not in connection with a pelvic examination. There was no real place for what we intuitively felt to be infinitely important, namely, supporting the women while confronting the emotions contained in their most private part of the body, the pelvis and its organs.

It gives pause for thought that there are alternative therapists who sell the service "vaginal acupressure", which is increasingly commonly practiced and accepted (in, for example, Denmark). Vaginal acupressors have made a living from massaging the acupressure points in the vaginas of women who typically suffer from diminished libido[44] and urine incontinence[45]. These treatments seem to be popular, they have surprisingly few reported side effects, but we believe that such procedures are best done by educated, trained, and supervised health professionals, preferably physicians.

It is clear that we as physicians are battling against our absolute terror of sex in society in general and in the entire health service in particular. We may conclude that when blocked or traumatized areas generally react positively to touch and the laying-on of hands, it is not so surprising that sexual areas do so too. As long as it is ensured that the patient is in full control, not violated, and that the therapist does not have sex with the patient in any form (in other words does not seduce her or manipulate her into a sexual relationship, which we see as "professional incest" and a criminal act that is not acceptable), we believe that such a treatment cannot be unethical. It is an important thing for a physician to be able to support his patients fully, including in the sexual sphere.

The subject of ethics has been of utmost importance to the physician since Hippocrates (460–377 BCE) and whenever the physician touches the patient, the ethics of the action must be considered. As often pointed out in the Hippocratic writings[12], the physician should have the healing of the patient as his sole focus. If the intention of the physician is wholehearted and rooted in deep medical expertise in order to heal the patient, his life and existence (and in this intention touch any part of the body including the genitals), then we believe the treatment is ethical. This kind of expertise is the expertise of the experienced holistic physician who can take his/her patient into the state of consciousness we know as the process of salutogenesis[20,21], or holistic existential healing[18,19]. We believe, as did Hippocrates, that the ethics of the physician seem to be proportional with his results with his patients[12]. Only the clearest of intentions can bring us outstanding results.

We believe that the technique of acupressure through the vagina, followed by existential conversation and further processing, is sufficient to induce the holistic healing of patient in the pelvic area and the sexual realm. The next logical step in our research is to take the holistic methods we have developed into controlled clinical testing, where we believe the square curve paradigm to be useful[59,60,61].

Let us end this discussion with a serious warning. Many (23%) incest victims felt that their therapist abused them sexually during therapy[62] and they often developed this feeling long after the therapy ended. This is presumably due to the mental reorientation necessary for rerepression of the painful emotions emerging during therapy, which in the end of therapy is not sufficiently integrated. It is extremely important to contact your patients sufficiently long after the closure of the therapy to be sure that the patient is not building this kind of idea, which will be harmful both to the patient and to the physician. As it is incest victims reporting this experience, it is extremely important that you process all incest traumas to the end before closing the therapy. It is important to make the patient agree to be in therapy for a sufficiently long time for this thorough integration to happen. We recommend that the therapy last for about 2 years, and we recommend the physician to follow up 5 years after the closure of therapy to prevent sudden accusations of sexual abuse.

## CONCLUSION

Pelvic massage, or acupressure through the vagina, is a bodywork technique. It seems to have been used by physicians ever since Hippocrates. It has been condemned as pornographic for a century, but it still seems to be very much needed to cure chronic patients suffering from a wide range of problems resistant to standard treatment and related to the structures of the pelvis: urine incontinence, tensions and chronic pains, a wide range of sexual problems.

The rationale behind acupressure though the vagina is that this procedure can help the patient to confront old painful emotions held by the local tissue and to identify and let go of negative beliefs and decisions from the traumatic life events. This procedure is a way to free the patients and their pelvis from the repressed feelings, love, and purpose of life. When the patients feel their repressed emotions, understand their message, and let go of the negative beliefs that have repressed them, they will heal their whole existence, including the body, its organs, energy, and sexuality at large.

The ethical aspects in holistic sexology are of extreme importance. As long as the physician loves and cares for his patient, gets the trust of the patient, gives holding flawlessly, and as long as it is ensured that the patient is in full control and not in any way violated, such a treatment can never be unethical. The physician must certainly follow the ethical rules of the country where the practice is performed and many countries have legal restrictions to such a holistic practice. It is important to understand that this contact is not and shall not be a sexual contact. The most important qualification of the physician trained in the bodyworks of holistic medicine is his/her ability to control his/her own intention and level of sexual excitement to ensure that this contact never turns into a sexual contact.

As one patient in three has some problem related to sex and gender, it is important for the holistic physician to be able to support his/her patients fully, including in the sexual sphere. Acupressure through the vagina seems to be a valuable tool for helping many women, but further research is needed to document its clinical value. In every pelvic examination there will be an element of acupressure through the vagina and the physician always needs to be aware of the repressed feelings that are released by this procedure. If the physician ignores the emotional pains re-experienced by the woman during the pelvic exam, this procedure will become more and more difficult and the woman will soon hate this examination, which unfortunately is a constant part of every modern woman's life. We, therefore, recommend that every physician be well acquainted with the method of acupressure through the vagina.

## ACKNOWLEDGMENTS

This study was supported by grants from IMK Almene Fond. The quality of life research was approved by the Copenhagen Scientific Ethical Committee under number (KF)V.100.2123/91 and later correspondence.

## BIOSKETCHES

**Søren Ventegodt, MD,** is the director of the Nordic School of Holistic Health and Quality of Life Research Center in Copenhagen, Denmark. He is also responsible for a Clinical Research Clinic for Holistic Medicine in Copenhagen and is a popular speaker throughout Scandinavia. He has published numerous scientific or popular articles and a number of books on holistic medicine, quality of life, and quality of working life. His most important scientific contributions are the comprehensive SEQOL questionnaire, the very short QOL5 questionnaire, the integrated QOL theory, the holistic process theory, the life mission theory, and the ongoing Danish Quality of Life Research Survey, 1991–94 in cooperation with the University Hospital of Copenhagen and the late professor of pediatrics, Bengt Zachau-Christiansen, MD, PhD. E-mail: ventegodt@livskvalitet.org. Website: www.livskvalitet.org

**Birgitte Clausen, RN,** leader of a secluded ward for people with dementia. Teacher at Nordic School of Holistic Medicine. Dementia coordinator and consultant in Aarhus, Denmark with more than 20 years of experience in adult psychiatry and dementia. During the last 4 years, she has worked together with Søren Ventegodt on the “QOL as medicine project” and on developing existential holistic group therapy. Through her articles and lectures, she has disseminated “The New Culture of Dementia” in both Denmark and Japan. She has participated in the production of several videos to order to inspire relatives, medical staff, and volunteers to understand people with dementia. This project has been granted financial support from the Ministry of Social Affairs in Denmark. She has conducted the first Danish investigation on the effect of sufficient daylight on the human spirit, activity level, and biological rhythm. E-mail: villahvide@mail.dk

**Hatim Omar, MD,** is Professor of Pediatrics and Obstetrics/Gynecology and Director of Adolescent Medicine and Young Parent programs at the Kentucky Clinic University of Kentucky in Lexington. Dr. Omar has numerous publications in child health, adolescent health, medicine, obstetrics, and gynecology. E-mail: haomar2@pop.uky.edu

**Joav Merrick, MD, DMSc,** is professor of child health and human development affiliated with the Center for Multidisciplinary Research in Aging, Zusman Child Development Center, Division of Pediatrics and Community Health at the Ben Gurion University, Beer-Sheva, Israel; the medical director of the Division for Mental Retardation, Ministry of Social Affairs, Jerusalem; and the founder and director of the National Institute of Child Health and Human Development. Dr. Merrick has numerous publications in the field of child health and human development, rehabilitation, intellectual disability, disability, health, welfare, abuse, advocacy, quality of life, and prevention and received the Peter Sabroe Child Award for outstanding work on behalf of Danish Children in 1985 and the International LEGO-Prize (“The Children’s Nobel Prize”) for an extraordinary contribution towards improvement in child welfare and well being in 1987. E-mail: jmerrick@internet-zahav.net. Website: www.nichd-israel.com
